# Compact Laser Doppler Flowmeter (LDF) Fundus Camera for the Assessment of Retinal Blood Perfusion in Small Animals

**DOI:** 10.1371/journal.pone.0134378

**Published:** 2015-07-30

**Authors:** Marielle Mentek, Frederic Truffer, Christophe Chiquet, Diane Godin-Ribuot, Serge Amoos, Corinne Loeuillet, Mario Bernabei, Martial Geiser

**Affiliations:** 1 HP2 laboratory, Grenoble Alpes University, 38000, Grenoble, France; 2 INSERM U1042 laboratory, 38000, Grenoble, France; 3 University of Applied Sciences and Arts of Western Switzerland, Institute of Systems Engineering, Sion, Switzerland; 4 Department of Ophthalmology, Grenoble University Hospital, Grenoble Alpes University, 38000, Grenoble, France; University of Louisville, UNITED STATES

## Abstract

**Purpose:**

Noninvasive techniques for ocular blood perfusion assessment are of crucial importance for exploring microvascular alterations related to systemic and ocular diseases. However, few techniques adapted to rodents are available and most are invasive or not specifically focused on the optic nerve head (ONH), choroid or retinal circulation. Here we present the results obtained with a new rodent-adapted compact fundus camera based on laser Doppler flowmetry (LDF).

**Methods:**

A confocal miniature flowmeter was fixed to a specially designed 3D rotating mechanical arm and adjusted on a rodent stereotaxic table in order to accurately point the laser beam at the retinal region of interest. The linearity of the LDF measurements was assessed using a rotating Teflon wheel and a flow of microspheres in a glass capillary. In vivo reproducibility was assessed in Wistar rats with repeated measurements (inter-session and inter-day) of retinal arteries and ONH blood velocity in six and ten rats, respectively. These parameters were also recorded during an acute intraocular pressure increase to 150 mmHg and after heart arrest (*n* = 5 rats).

**Results:**

The perfusion measurements showed perfect linearity between LDF velocity and Teflon wheel or microsphere speed. Intraclass correlation coefficients for retinal arteries and ONH velocity (0.82 and 0.86, respectively) indicated strong inter-session repeatability and stability. Inter-day reproducibility was good (0.79 and 0.7, respectively). Upon ocular blood flow cessation, the retinal artery velocity signal substantially decreased, whereas the ONH signal did not significantly vary, suggesting that it could mostly be attributed to tissue light scattering.

**Conclusion:**

We have demonstrated that, while not adapted for ONH blood perfusion assessment, this device allows pertinent, stable and repeatable measurements of retinal blood perfusion in rats.

## Introduction

Laser Doppler flowmetry (LDF) is a technique widely used to assess red blood cell (RBC) perfusion in tissues. In 1972, Riva et al. first described the Doppler effect for the evaluation of retinal perfusion in rabbits [1]. Since then, several LDF prototypes have been developed in animals to evaluate blood perfusion in various vascular beds of the eye, such as the retina, the optic nerve head (ONH) and the choroid [2]. These prototypes were developed for a range of different species, such as primates [3], rabbits [4], cats [5], birds [6] and minipigs [7].

The rat has been extensively studied in the fields of physiology, physiopathology and therapeutics. Researchers have access to different rodent models of systemic (diabetes, hypertension) and ocular (glaucoma, diabetic retinopathy, nonarteritic ischemic optic neuropathy, hereditary optic nerve and retinal) diseases. For rats, noninvasive techniques of ocular blood perfusion measurement have previously been developed, including LDF [8], laser speckle [9], optical coherence tomography (OCT) [10], optical microangiography (OMAG) [11], magnetic resonance imaging (MRI) [12], the scanning laser ophthalmoscope-particle tracking method [13] and scanning LDF [14]. Many of these techniques require specific and expensive equipment for animal studies (OCT, MRI, OMAG) and highly qualified operators. Other techniques of blood perfusion measurements are invasive, using a LDF probe either inserted into the vitreous cavity [15] or placed on the sclera after conjunctival dissection [16]. Finally, some techniques require the sacrifice of the animal such as those using (^14^C)-iodoantipyrine [17], N-Isopropyl-p- [^14^C]-iodoamphetamine [^14^C]-IMP tracer [18] or radiolabeled microspheres [19].

LDF [2] is based on the detection of the Doppler shift of light scattered by a moving particle. This technique provides relative and repeatable values of choroidal and ONH blood perfusion in humans in real time [20, 21]. Given the extensive use of rodents in physiology and therapeutics, the development of a similar LDF technique for the study of blood perfusion in the vascular beds of the rat eye (retina, ONH and choroid) is desirable.

For these reasons, we aimed to develop a compact, inexpensive and noninvasive LDF instrument for repeatable measurements of rodent ocular blood perfusion. This device has been particularly designed for repeated measurement of retinal and optic nerve head blood flow over time. Technical data and extensive measurements in rats are therefore reported herein.

## Material and Methods

### Optical system description

**Fig 1:**
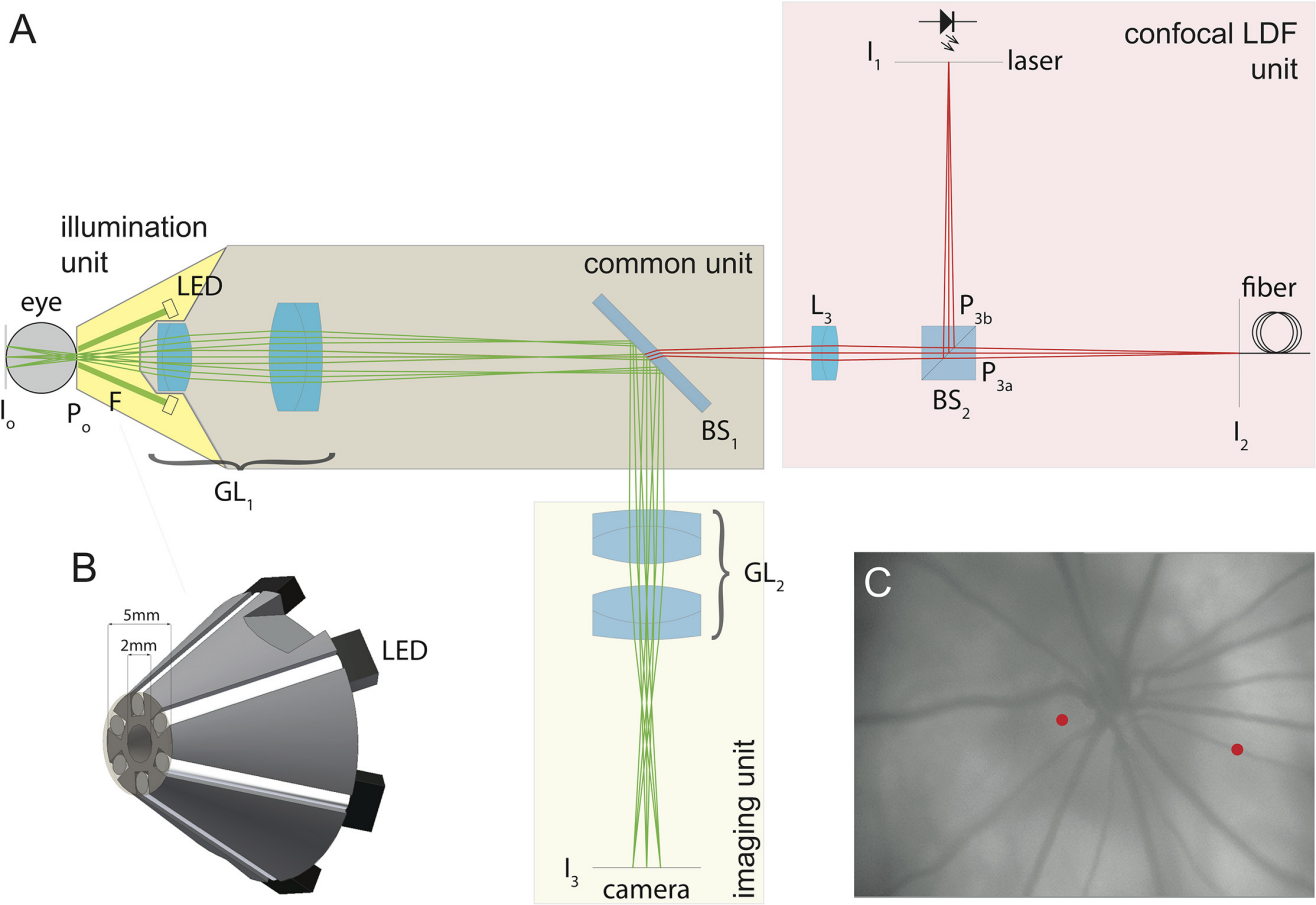
LDF optical unit scheme. (A) Optical system with the different optical units. I_n_ are conjugated images and P_n_ are conjugated pupils. F: illumination fiber; GL_1_: first relay with focusing; BS_1_: cold mirror; L_3_: relay lens; BS_2_: beam splitter cube with pupil images on surfaces; I_1_ laser source and I_2_ detection fiber; GL_2_: relay lens group to CCD camera at I_3_. (B) 3D view of the front part of the instrument with the glass rods and LEDs to illuminate the fundus. Central hole of 2 mm acts as entrance pupil (P0). (C) Fundus image obtained with the imaging unit. Dots represent typical LDF recording locations on the ONH and retinal artery.

The optical system, designed for Campbell and Hughes’s general rat eye model [22] consists of an external illumination source, a common unit, an imaging unit and a confocal LDF unit [23] (Fig 1).

The common unit images the fundus (I_o_) with GL_1_ onto an intermediate image (I_o′_), which is relayed by the confocal LDF and imaging units. The focus is adjusted with the front lens of GL_1_ along the optical axis. A cold mirror (BS_1_) divides the optical paths between the imaging and LDF units. In this later unit are two images (P3a, P3b) of the pupil of the rat (P0).

An annulus of six glass rod ends, with a protective 125 μm thin glass plate to prevent dust and liquid entering the system, delivers up to 830 μW of light into the eye (front illumination unit). On the other end of each glass rod, a color filter (central wavelength, 530 nm; Edmund Optics, Karlsruhe, Germany) and a white LED (LW P4SG-U2AA-5K8L-Z, Osram, Munich, Germany) are glued.

The imaging unit (GL_2_) focuses the light onto a CCD camera with 640×480 active pixels (20K13XUSB, Videology, The Netherlands). Visualization of the retina and projection of the laser onto the target are provided by the camera (Fig 1). The field of view, limited by the size of the CCD sensor to 28×20 degrees in air, corresponds to a 1.3 magnification.

The LDF unit is built on a confocal arrangement (Fig 1). The probing laser light I_1_ (780 nm, 5 mW, Thorlabs, Munich, Germany) goes through a 50/50 beam splitter (BS_2_) and enters the eye with an adjustable power of 300, 400 or 500 μW, below the reported minimum light intensity known to cause phototoxic effects on rat photoreceptors [24]. The laser beam is focused onto the point of interest on the rat retina (estimated diameter, 10 μm). Some light is backscattered by the red blood cells (RBCs) with a Doppler shift proportional to their speed. Light reflected by the tissue, mixed with that scattered from the RBCs, is collected by the optical system and directed towards an optical fiber I_2_ (200 μm diameter; Thorlabs, Munich, Germany). The latter guides the light to an avalanche photodiode (C5460-01, Hamamatsu, Japan). The portion of the ocular fundus probed has a diameter of about 80 μm, meaning that I_2_ collects light diffused around the illumination point.

### Data acquisition and software description

Due to the optical arrangement and blood velocity, the maximum Doppler shift to be acquired is set at 5 kHz. This signal is Fourier-analyzed 14 times per second. Direct current (*DC*) corresponds to the intensity of the collected light. The blood perfusion parameters (velocity, *Vel*) and (volume, *Vol*) were calculated as the first moment of the power spectrum and the *DC* ^*2*^ normalized integral of the power spectrum, respectively. The weighted mean *Vel* (expressed in Hz) gives a frequency shift proportional to RBC velocity. The signal analysis was previously described [23]. *DC* is used to estimate the stability of the eye and the light scattering. In addition, the eye fundus is continuously observed using the imaging unit during measurements, and fundus images are captured every 30 s to permit qualitative evaluation of eyeball stability.

### Mechanical system

**Fig 2:**
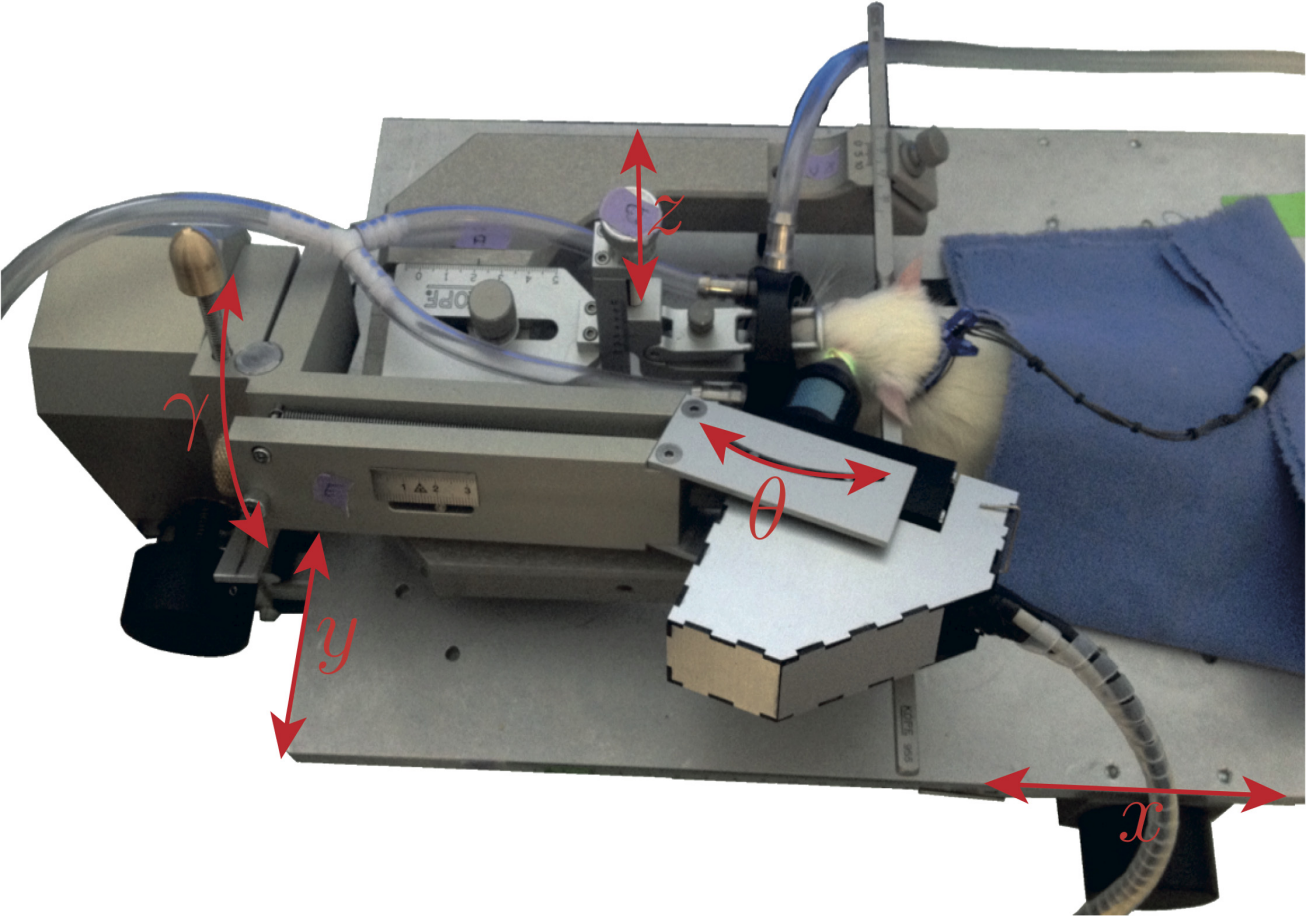
Stereotaxic table view. The LDF unit is held in three dimensions by a rotating arm. Accurate movements are enabled by five micrometric screws. *x* and *y* permit movements of the table in the horizontal plane. *z* permits vertical movements of the rat head. Θ and γ permit modification of the angulation of the emitted light into the eye.

The rat is fixed on a modified stereotaxic instrument (Kopf instruments, Tujunga, CA, USA). The optical device is mounted on a dedicated positioning holder (Fig 2), which allows an adjustment in the (x, y, z) directions to bring the pupil of the optical device into the pupil of the eye, with 10-μm precision, without exerting any pressure on the eye. Then a rotation (θ, γ) around the center of the animal’s pupil brings the probing beam to the desired retinal location.

### Animal preparation

#### Ethics statement

All experiments were conducted on adult male Wistar rats (8 weeks old; weight range, 300–330 g; Janvier Labs, France). The experiments were conducted in strict accordance with the European Convention for the Protection of Vertebrate Animals used for Experimental and Other Scientific Purposes (Council of Europe, European Treaties ETS 123, Strasbourg, 18 March 1986) and with the Guide for the Care and Use of Laboratory Animals of the National Institutes of Health (Publication No. 85–23, revised 1996). The protocol was approved by the Committee on the Ethics of Animal Experiments of Grenoble Alpes University (Comité d’éthique ComEth Grenoble, permit number: 38 10 42). The rats were housed under diurnal lighting conditions and given free access to food and water. All surgery was performed under general anesthesia, and all efforts were made to minimize suffering.

#### Anesthetic protocol

The animals were anesthetized with buprenorphine (0.1 mg/kg, subcutaneously, Buprecare, Axience, France), xylazine (6 mg/kg, intramuscular injection, Rompun, Bayer, France) and diazepam (5 mg/kg, intraperitoneal injection, Valium, Roche, France). This protocol has been designed to ensure minimal cardiovascular depression and optimal eye stability. The anesthetic protocol has been modified from previously described one [25] and assessed under veterinary control. Unconsciousness and loss of deep sensibility were controlled during the experiment by frequent assessment of deep sensitivity (every 5 min) by pinching the foot with a forceps (pedal reflex), and by observation of a stable heart rate and eye globe position. Animals were intubated and mechanically ventilated (75 breaths/min, tidal volume of 1.5 ml) using a rodent respirator (model 683, Harvard Apparatus, MA, USA). Oxygen saturation (MouseOx Mouse Oxymeter, STARR Life Sciences Corporation, USA), heart rate and mean arterial blood pressure (Powerlab, ADInstruments, Oxford, UK) were monitored continuously. Body temperature was monitored and maintained at 37–38°C using a homoeothermic plate (Physitemp Instruments, NJ, USA).

The animals were placed in ventral recumbence on the stereotaxic table. The head was maintained by auricular bars to ensure stability and safety of the vertebral column (Fig 2).

The pupil was dilated with 1% tropicamide (Mydriaticum, Théa, Clermont-Ferrand, France) and the cornea was anesthetized by instillation of oxybuprocaine chlorohydrate (Théa, Clermont-Ferrand, France). Ophthalmic gel (Lacrigel, Europhta, Monaco) was used to prevent the cornea from drying during the measurements and to provide better image quality by reducing aberrations.

The perfusion parameter velocity in retinal arteries (*Vel*_*ART*_) was measured at a distance of 0.5–1 papillary diameter from the border of the ONH. The same parameter within the ONH tissue (*Vel*_*ONH*_) was measured on the neuroretinal rim, between major retinal vessels (Fig 1B).

### Experiments

#### Linearity of LDF measurements in simulation experiments

The linearity of LDF measurements was first confirmed using a Doppler shift simulator, which consisted of a rotating Teflon wheel [20], on which the probing beam was focused. In another series of measurements, the probing light was focused on a simulated retinal vessel consisting of a glass capillary tube (200 μm in diameter) in which latex microspheres (1 μm in diameter, Polysciences Europe GmbH, Eppelheim, Germany), suspended in water, were moved in a laminar flow using a syringe pump. The speed of the wheel and the microspheres was changed stepwise. Perfusion parameters were averaged over 40-s LDF recordings. With these simulations, Vel should vary linearly with the speed of the wheel and of the flowing microspheres, whereas Vol should remain constant.

#### Repeatability of LDF measurements in rats

Intra- and inter-session Vel_ART_ and Vel_ONH_ signal repeatability was assessed on day 1 (D1) by three consecutive 5 min recordings separated by 10 min intervals (n = 6 and 10 rats, respectively). Eye stability was evaluated afterwards by image analysis (Image J software, National Institutes of Health, Bethesda, MD, USA) and stereotaxic micrometric coordinates were recorded for each measurement location. At the end of the experiment, rats were awakened by intramuscular injection of atipamezole (0.1 mg/kg, Antisedan, Orion Pharma, France). They underwent close monitoring in the next hours and were checked for any post-anesthetic complications during the following days. Housing conditions did not vary between D1 and D8.

One week later (D8), the animals underwent the same anesthetic protocol, and inter-day repeatability was assessed by a single 5 min recording of *Vel*_*ART*_ and *Vel*_*ONH*_ performed at the same locations as on D1. At the end of the experiment, the animals were sacrificed with an intravenous pentobarbital sodium (Ceva, France) overdose.

We also quantitatively evaluated the precision of the laser beam repositioning using the stereotaxic micrometric coordinates and a Malassez cell as a target. The laser beam was directed on day 1 at a point in the micrometric grid of the Malassez cell and the precise position of each micrometric screw was carefully recorded. The beam was then randomly redirected away from the Malassez cell. On day 2, the laser beam was repositioned using only the micrometric screw positions. Deviation of the target point between day 1 and day 2 was recorded for five different locations on the Malassez cell.

#### Transient ocular blood flow changes

In six rats, the anterior chamber was cannulated with a 27G^1/2^ needle connected to a reservoir filled with balanced salt solution (Baush and Lomb, Rochester, NY, USA) and to a pressure transducer (Edwards Lifesciences, Guyancourt, France). Intraocular pressure (IOP) was continuously monitored (Powerlab, ADInstruments, Oxford, UK) and modified by increasing the height of the bottle in order to reach an IOP of 150 mmHg. LDF measurements were taken for 2 min at baseline IOP (physiological IOP, 15 mmHg), followed by 4 min at 150 mmHg and finally by 5 min at baseline. For each rat, four recordings (on two retinal arteries and two ONH locations) were performed with a resting period of 20 min between them. At the end, the animals were sacrificed and Vel_ART_ was recorded continuously upon heart arrest and Vel_ONH_ was recorded for 5 min after heart arrest. All recordings were performed in the same retinal or ONH locations throughout the IOP experiment.

### Statistical analysis

Data processing was performed using R [26] and statistical analysis using the Statistical Package for the Social Sciences (SPSS; version 17.0, SPSS Inc., Chicago, IL, USA). *P* < 0.05 was considered statistically significant. All tests were two-tailed. Data are expressed as mean ± standard deviation. Statistical comparison during baseline, ocular hypertension and after heart arrest was performed using nonparametric Wilcoxon signed-rank tests. The correlation of the ocular blood perfusion parameters with the speed of the Teflon wheel and of the microspheres was assessed using Pearson tests. Repeatability of the measurements was assessed between each minute of the 5 min measurements (intra-session), between the three repeated 5 min measurements (inter-session) and between the first recording on D1 and D8 (inter-day). Intra- and inter-session, and inter-day repeatability of *Vel*_*ART*_ and *Vel*_*ONH*_ were estimated by calculating the variation coefficients (VC) and the Intraclass Correlation Coefficient (ICC) (single, two-way random absolute agreement). ICC values were interpreted as follows: 0–0.49 indicating poor agreement, 0.5–0.69 indicating fair agreement and >0.7 indicating good to strong agreement.

## Results

### Linearity of LDF measurements in simulation experiments

**Fig 3:**
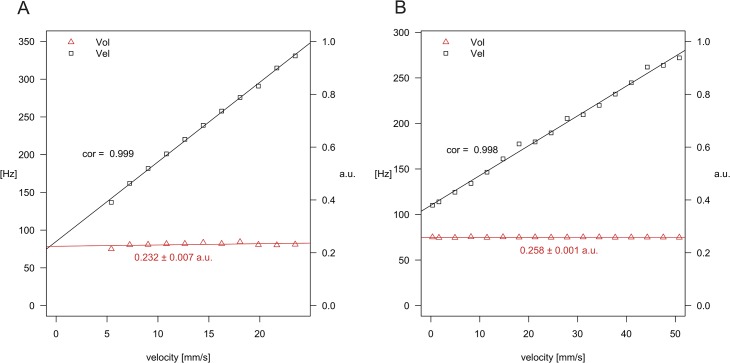
Linearity assessment of LDF parameters. Ocular blood perfusion parameters *Vol* (volume) and *Vel* (velocity) versus the velocity obtained from a rotating Teflon (A) wheel and a flow of microspheres in a glass capillary (B).

The results of the LDF measurements recorded using the Teflon wheel and latex microspheres are shown in Fig 3. *Vel* varied linearly with the velocity of the wheel (Pearson correlation factor, 0.999, *p* < 0.0001). *Vol* remained constant over the range of induced velocity (0.232 ± 0.007 a.u., Fig 3A). Due to the minimum voltage required to operate the motor, velocities lower than 5 mm/s could not be tested. Similar results were obtained using flowing microspheres: *Vel* varied linearly with the velocity of the microspheres in the capillary (Pearson correlation factor, 0.998, *p* < 0.0001). *Vol* remained constant (0.258 ± 0.001 a.u., Fig 3B). *DC* (data not shown) remained constant in both setups over the entire velocity range (Teflon wheel 0.359 ± 0.001 Volt and microspheres 0.338 ± 0.004 Volt).

### Baseline velocity values in physiological conditions

Cardiovascular monitoring of each rat during anesthesia showed stable mean arterial blood pressure and heart rate, and physiological blood oxygen saturation (122 ± 3 mmHg, 332 ± 5 beats/min and 98.7 ± 0.6%, respectively). During baseline measurements in retinal arteries and ONH, *Vel*_*ART*_ and *Vel*_*ONH*_ were 651 ± 76 Hz (*n* = 12 rats) and 369 ± 138 Hz (*n* = 16 rats), respectively.

The mean spectra from retinal arteries were characterized by a power intensity distribution over a wide range of frequency shifts (30 to 3000–4000 Hz), whereas the mean spectra from ONH had a power intensity distribution over a lower range of frequency shifts (30–1000 Hz). The mean spectra from tissue proximal to the investigated retinal vessels (i.e., choroid) were similar to the ONH spectra.

### LDF signal repeatability

After analysis of ocular fundus images for all retinal and ONH measurements, we excluded one 5-min measurement for *Vel*_*ART*_ evaluation on D1 in two rats because of eyeball movements.

**Fig 4:**
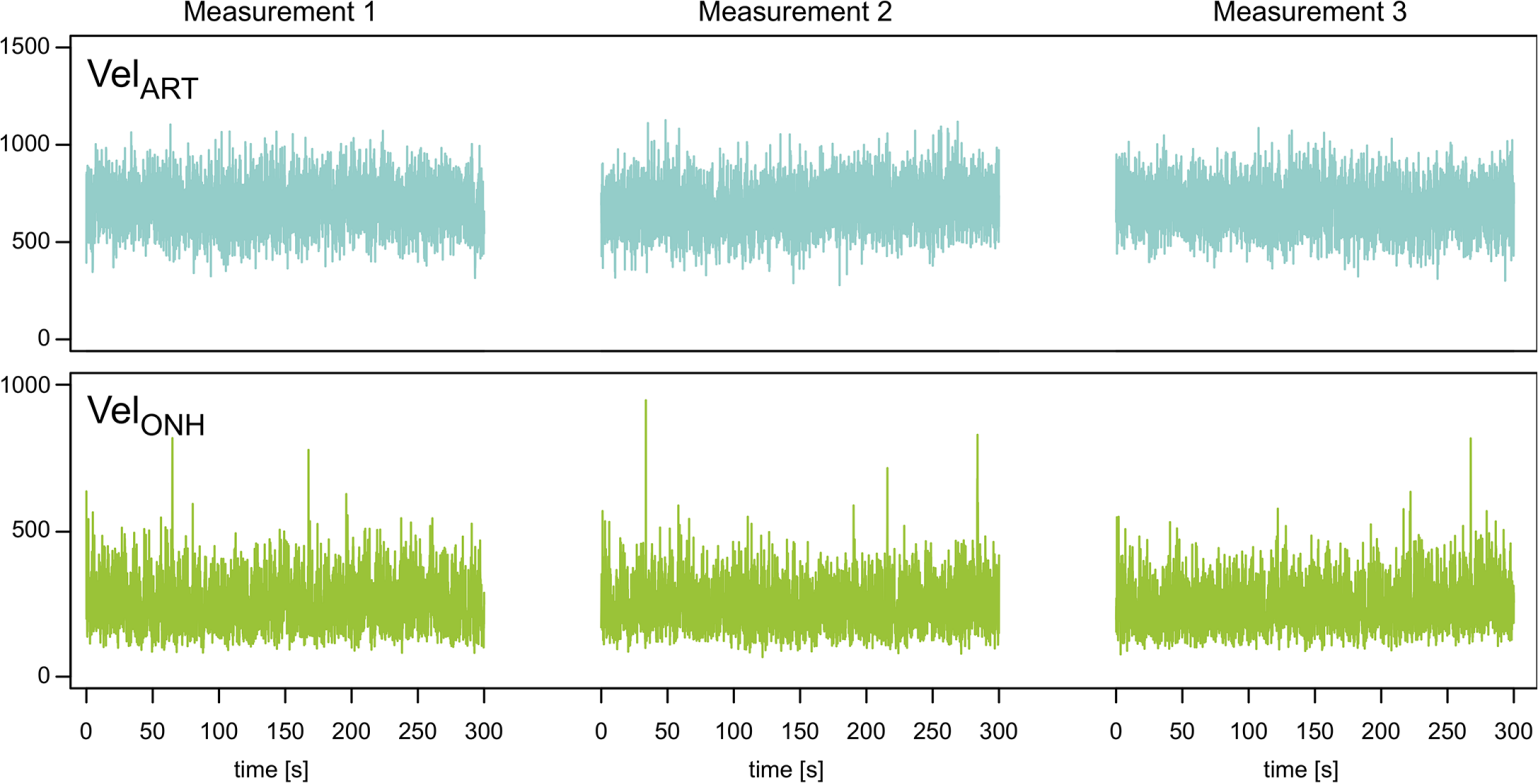
Repeated velocity measurements. Example of *Vel*_*ART*_ and *Vel*_*ONH*_ recordings repeated every 5 min for one rat.

The repeatability of the measurements (variation coefficients and ICC) is reported in Table 1. *Vel*_*ART*_ and *Vel*_*ONH*_ repeatability is illustrated in Fig 4 with representative examples of repeated measurements on D1.

**Table 1:** Intraclass correlation coefficient and variation coefficient for retinal artery and optic nerve head velocity. For Intra-session evaluation, each 5-min measurement was divided into five 1-min periods. Mean velocities in each 5-min or 1-min session were considered. *P*-value and 95% confidence interval are given for each corresponding ICC value.

	Velocity, retinal arteries					Velocity, optic nerve head				
			95% confidence interval					95% confidence interval		
	VC (%)	ICC	lower bound	upper bound	p	VC (%)	ICC	lower bound	upper bound	p
Intra-session	3.6±3.4	0.4	0.12	0.91	<0.05	9.2±4.2	0.6	0.4	0.84	<0.05
Inter-session	7±5.1	0.82	0.29	0.98	<0.05	8.1±7	0.87	0.68	0.96	<0.05
Inter-day	7.8±5	0.79	-0.22	0.98	<0.05	7.6±9	0.7	-0.3	0.97	0.07

*Vel*_*ART*_ and *Vel*_*ONH*_ were highly repeatable, with inter-session ICCs of 0.82 and 0.86, respectively, indicating strong agreement. However, intra-session ICCs were 0.4 and 0.6, respectively, reflecting the high short-term variation of the *Vel* signal (1 min).

The accuracy of the laser beam repositioning on the pupil, assessed using the Malassez cell, was considered satisfactory in most cases, with a median 50 μm difference between the two consecutive positions on day 1 and day 2.

The five different positions on day 2 were respectively 20, 50, 75, 20 and 100 μm away from those of day 1.

Concomitantly, the inter-day ICC of 0.79 for *Vel*_*ART*_ reflected the accuracy of laser beam repositioning, while inter-day ICC for *Vel*_*ONH*_ did not reach statistical significance.

### Effects of IOP variations

**Fig 5:**
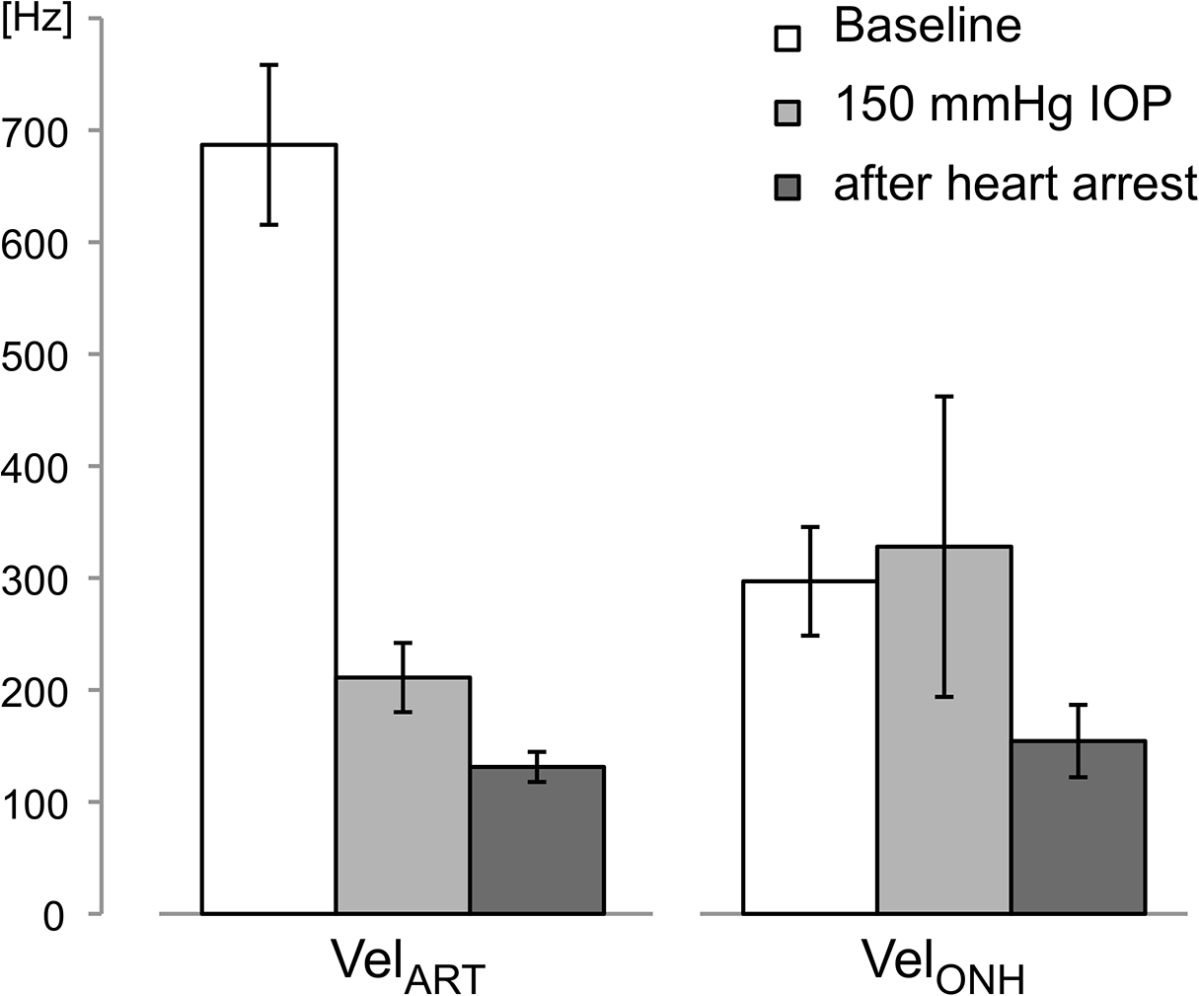
*Vel*_*ART*_ and *Vel*_*ONH*_ variations in response to 150 mmHg and after heart arrest (mean ± SD).

**Fig 6:**
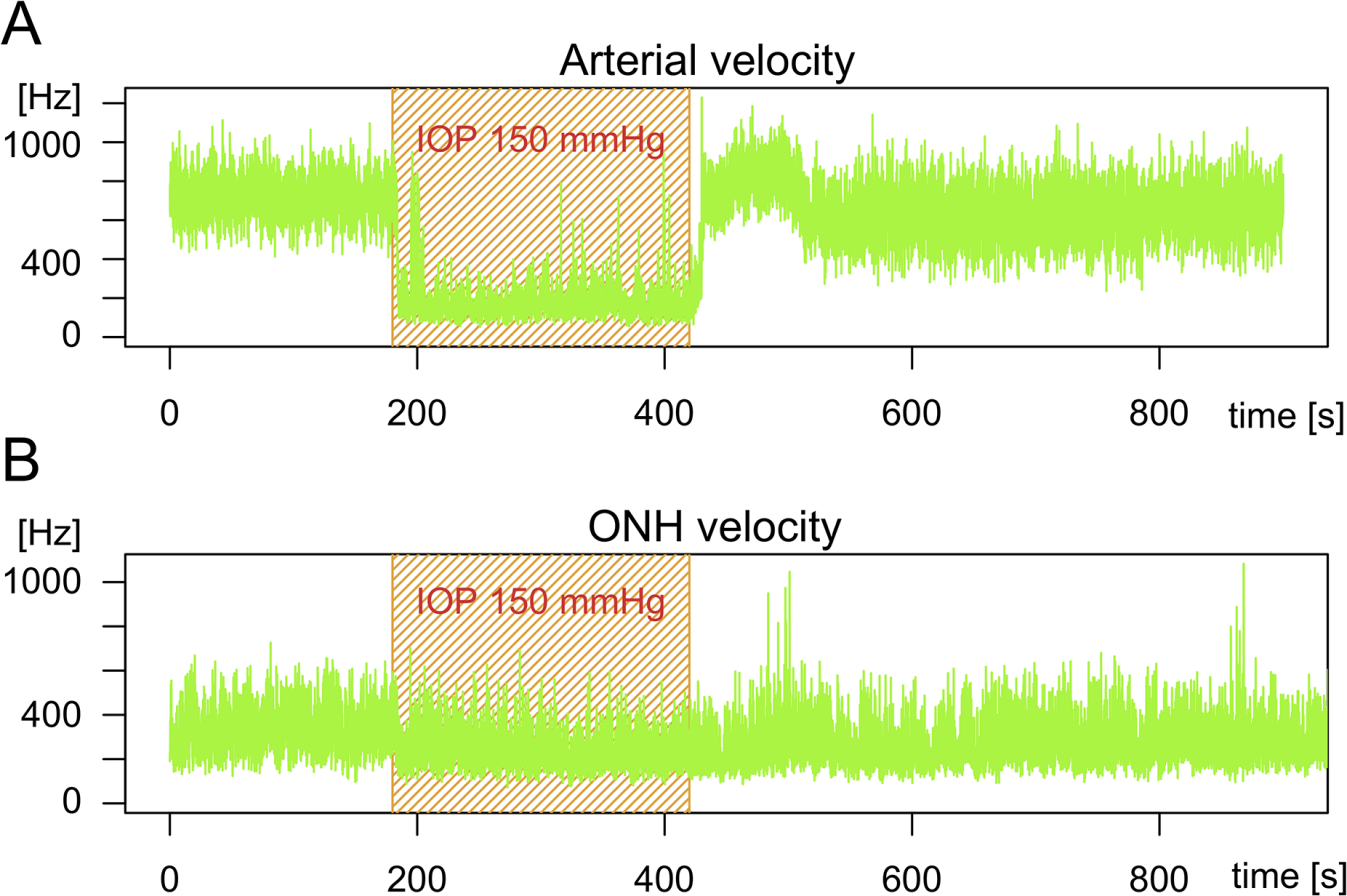
Intraocular hypertension experiment results. Typical recordings of retinal arteries (A) and optic nerve head (B) velocity during intraocular hypertension experiment. After 3 min of baseline recording (IOP, 15 mmHg), IOP was increased to 150 mmHg for 4 min and returned to 15 mmHg for the end of the measurement (recovery period).

An increase in the IOP value to 150 mmHg decreased *Vel*_*ART*_ by 69 ± 9% (*p* = 0.04) and *Vel*_*ONH*_ by 4.5 ± 19.5% (*p* = 0.39) compared to baseline values (Fig 5). Fig 6 illustrates the real-time changes in *Vel*_*ART*_ and *Vel*_*ONH*_ in the response to intraocular hypertension.

**Fig 7:**
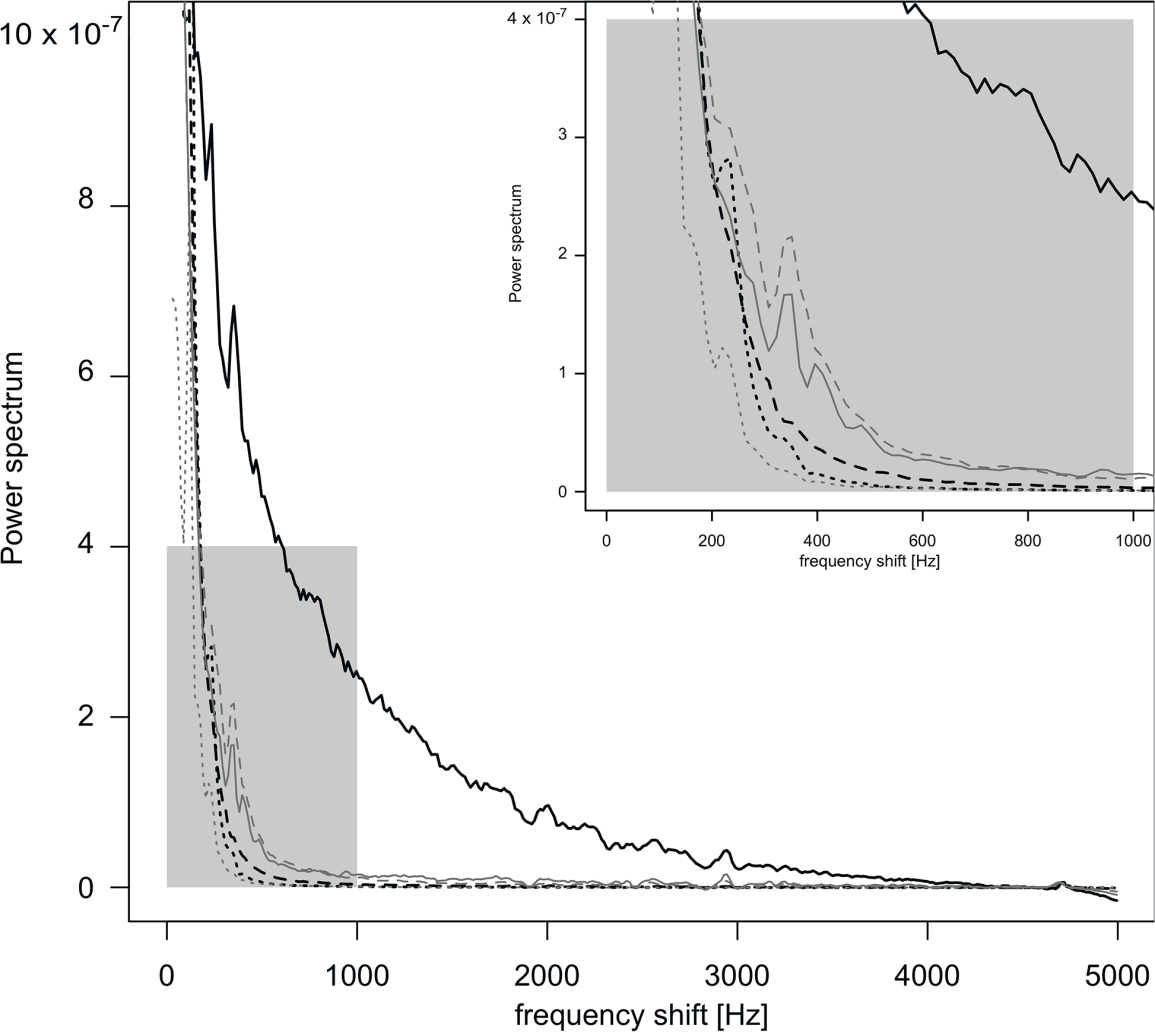
Power spectra analysis during the three experimental conditions. Variation of power spectra at baseline, during the IOP experiment and after heart arrest, for velocity of retinal artery (black lines) and optic nerve head (gray lines). Each period of the experiment is represented by the corresponding mean power spectrum: baseline (solid line), increased IOP at 150 mmHg (dashed line) and after heart arrest (dotted line). Upper right corner of the figure represents zoomed power spectra between 0 and 1000 Hz (gray area).

After heart arrest, *Vel*_*ART*_ and *Vel*_*ONH*_ were decreased by 81 ± 3% (*p* = 0.04) and 41 ± 15% (*p* = 0.04), respectively, compared to previous values. Fig 7 shows representative power spectra during baseline, intraocular hypertension and immediately following heart arrest.

## Discussion

We have developed a new compact LDF device dedicated to the study of ocular blood perfusion in the rat. The perfusion measurements obtained using this new prototype show a perfect linearity between measurements of *Vel*, Teflon wheel speed and microsphere speed. This LDF device estimated the mean spectra of the Doppler shift for the ONH tissue and retinal arteries in baseline conditions. Inter-session and inter-day reproducibility of blood velocity measurements were better than intra-session repeatability. The analysis of spectra and blood perfusion parameters during the IOP increase and after heart arrest validate the pertinence of the velocity signal from retinal arteries and strongly suggest that the *Vel*_*ONH*_ signal is mostly attributed to tissue light scattering.

The first step of validation of this new LDF device was to test the linearity of LDF measurements with a Teflon wheel and microspheres in a glass capillary. Due to the scattering process, the number of backscattered photons is known to be independent of the speed of the wheel so that the power spectrum integral, normalized with *DC* ^2^ (*Vol*), should be constant. As expected, in both experiments, *Vol* was constant and independent of speed. *Vel* measured with moving microspheres was linear over a much wider range (0.3–50 mm/s) than needed for the expected speed of RBC in the rat fundus (24 mm/s in retinal arteries [11]). The range of speed for the wheel is low because multiple scattering within Teflon induces a higher frequency shift than the software can accept. Therefore, the new LDF device shows the same linearity and similar spectra as described for the previous LDF systems used in humans [20].

Three parameters may be used to ascertain the quality of LDF measurements: stability of *DC* [23], control of eye stability and spectrum characteristics. DC, which corresponds to the intensity of the collected light, is monitored during LDF measurements to validate a stable measurement volume. It is the first parameter to vary in response to eyeball movements because sampled tissue volume, and therefore scattered light, will change. In our experiments, we were able to observe real-time eyeball movements, confirmed by fundus images captured every 30 s. These observations suggest that some very low amplitude movements of the eye globe do not affect *DC* stability but can induce *Vel* variations. We used post-measurement analysis of ocular fundus images as a qualitative index of ocular stability, permitting us to validate *Vel* recordings. This method allows us to exclude *Vel* variations due to slow ocular motion. Consequently, a quantitative method for fundus image analysis, and thus for eye motion evaluation, using image tracking, would be valuable in the near future.

Besides *DC* and fundus images, measurement validation incorporates the mean spectrum as a third quality criterion. The mean power spectrum for frequencies between 400 and 500 Hz should be higher than mean noise, which is calculated for frequencies above 4 kHz. Retinal arteries and ONH spectra showed different profiles, with lower frequency shift in ONH spectra. This is in accordance with the lower volume of RBCs in ONH tissue compared to retinal arteries.

Perfusion measurements are affected by the optical scattering properties of the tissue and by the heterogeneity of the distribution of capillaries. Measurements show that retinal blood vessels do not affect ONH perfusion recording when they are at a distance greater than 100 μm from the measurement area in the ONH (data not shown). In the case of *Vel* measurement in the retinal vessel, which has a diameter of approximately 50 μm [27, 28], most of the signal originates from RBCs in the vessels and the contribution of surrounding tissues was considered to be negligible.

Based on the ICC values discussed in the results section, reproducibility was considered good to strong for *Vel*_*ART*_ and *Vel*_*ONH*_. Inter-session reproducibility was higher than intra-session reproducibility. Short-term variations (1 min) of the signal were probably associated with eyeball movements, the main parameter affecting *Vel*_*ART*_ stability. We observed very stable eyeballs for some rats, whereas others showed eye motions in spite of the same anesthetic protocol. For inter-day reproducibility, which showed good to strong agreement for *Vel*_*ART*_ and *Vel*_*ONH*_, the positioning of the LDF was critical and improved by the modified stereotaxic instrument and the LDF holder, which allows adjustment in the (x, y, z) directions to bring the pupil of the optical device into the pupil of the eye with a precision of 10 μm (as shown using the Malassez cell). Owing to this setup, recording of blood perfusion in the area of interest is possible on different days, in order to follow blood perfusion over time.

An increase in IOP up to a value of 150 mmHg led to a major decrease in *Vel*_*ART*_. Complete cessation of retinal perfusion is visible with funduscopy (rapid disappearance of retinal vessels, whitening of the fundus) [29, 30] and previous experiments in rats have shown that an IOP as high as 130 mmHg strongly reduces blood perfusion [31, 32] in the choroid, retina and ONH. This IOP increase gave similar results in other species and using other measurement techniques (monkeys [33] and cats [34, 35]). Since mean arterial pressure during our experiments was less than 130 mmHg, ocular perfusion pressure at an IOP of 150 mmHg, estimated in the rat by the difference between mean arterial pressure and IOP, is expected to be close to zero. We can assume that the *Vel*_*ART*_ decrease is directly correlated to the retinal vessel diameter reduction and obstruction, as previously imaged in OCT [32] and as observed in ocular fundus images during our experiments. A similar decrease in ocular perfusion was observed after heart arrest. Mean spectra analysis showed a significant drop in intensity at frequencies over 200 Hz. This means that the number of moving RBCs (proportional to the area under the curve) drops significantly. These observations led us to conclude that the LDF signal recorded in retinal arteries is highly sensitive to an acute increase in IOP and reflects the decrease in blood perfusion in both conditions.

Contrary to *Vel*_*ART*_ changes during the IOP increase, *Vel*_*ONH*_ showed a nonsignificant decrease in response to ocular blood flow arrest. If this response reflects the absence of change in ONH blood flow, a possible explanation is that the ONH tissue sampled by LDF was significantly deeper than ONH tissues affected by the IOP increase and still perfused under these conditions [36]. The prelaminar and laminar ONH region of rats, about 170 μm thick, is likely to have been measured by LDF. Fredriksson et al. explored LDF measurement depth and volume in various tissues for a similar LDF probe setup. From these results, the depth of measurement for our device is estimated between 100 μm and 270 μm [37]. Moreover, comparison of the power spectrum obtained during intraocular hypertension and after heart arrest shows similar signals. These observations support the theory that Doppler shift induced by tissue light scattering contributes more to the *Vel*_*ONH*_ signal than RBC movements. This hypothesis is reinforced by the paucity of capillaries in the anterior part of the ONH sampled by our LDF device. An abundant capillary network irrigates the retrobulbar part of the optic nerve, but few capillaries have been observed in the superficial ONH layers [38, 39]. One scanning electron micrograph of a microvascular cast [40] shows that three to six capillaries are visible in the LDF measurement area. Other ocular blood perfusion measurement techniques such as microsphere velocity measurements show that perfusion in the rat ONH (0.18 ± 0.03 μl/min) was lower than that measured in the retina (19 ± 3.4 μl/min) and the choroid (170 ± 35 μl/min) [41]. These data strongly suggest that noise produced by light scattering in ONH tissue is responsible for the majority of the LDF signal.

There are some limitations in the use of our LDF device. It does not provide absolute perfusion values and therefore it is not possible to compare baseline perfusion between animals. Adding a rotating pupil with two scattering directions of detection and image analysis to achieve accurate measurement of vessel diameter would improve the ability of the device to measure the absolute flow in rat retinal vessels. Moreover, the absence of a pertinent signal coming from the ONH strongly suggests that this device is not useful for the study of ONH perfusion in rats.

This device should be considered as a new tool to assess retinal perfusion along with the other devices recently reported: LDF to assess global ocular perfusion [8], an invasive method to acquire perfusion information [16] and the promising but less affordable OCT-OMAG technique [11].

## Conclusion

We have described a new, compact LDF device dedicated to studying ocular blood perfusion in the rat allowing repeatable measurements on retinal vessels. The perfusion measurements obtained using this new prototype showed perfect linearity between measurements of *Vel* and speed of the Teflon wheel and microspheres. The mechanical system allows rotation of the lens through the rat’s pupil, providing easy targeting of retinal vessels. Regular recording of images and subsequent analysis can track the eye position and *a posteriori* selection of collected data. Estimation of the mean Doppler shift spectra for retinal arteries in baseline conditions, during IOP increase and after heart arrest validated the pertinence of the velocity signal from retinal arteries.

## Supporting Information

S1 File NC3Rs ARRIVE Guidelines Checklist 2014. (DOCX)
