# Supplementary material for: Compact Laser Doppler Flowmeter (LDF) Fundus Camera for the Assessment of Retinal Blood Perfusion in Small Animals
Source: PLoS One. 2015 Jul 30;10(7):e0134378. doi: 10.1371/journal.pone.0134378 (PMC4520556; doi:10.1371/journal.pone.0134378)

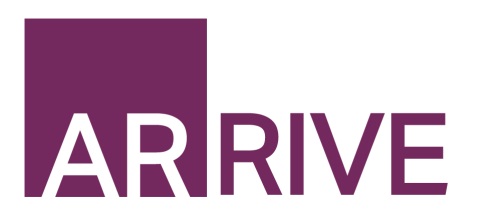


The ARRIVE Guidelines Checklist

Animal Research: Reporting In Vivo Experiments

Carol Kilkenny^1^, William J Browne^2^, Innes C Cuthill^3^, Michael Emerson^4^ and Douglas G Altman^5^

*^1^The National Centre for the Replacement, Refinement and Reduction of Animals in Research, London, UK, ^2^School of Veterinary Science, University of Bristol, Bristol, UK, ^3^School of Biological Sciences, University of Bristol, Bristol, UK, ^4^National Heart and Lung Institute, Imperial College London, UK, ^5^Centre for Statistics in Medicine, University of Oxford, Oxford, UK.*

|  | | ITEM | RECOMMENDATION | Section/ Paragraph |
| --- | --- | --- | --- | --- |
| 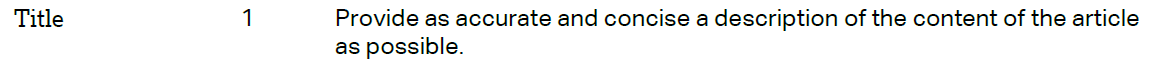 | | | page 1 |  |
| 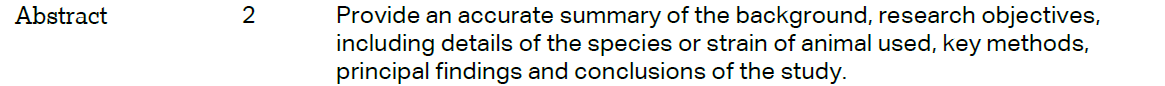 | | | page 2 |  |
| INTRODUCTION | | |  |  |
| 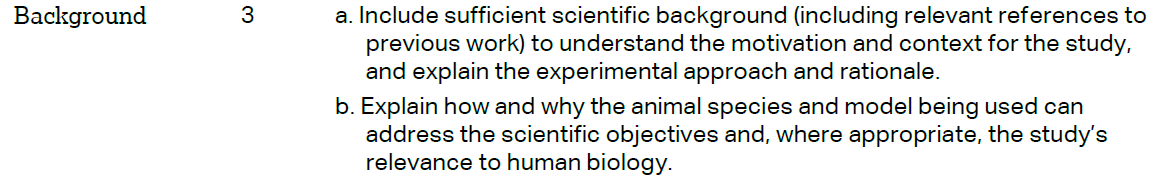 | | | page 4 |  |
| 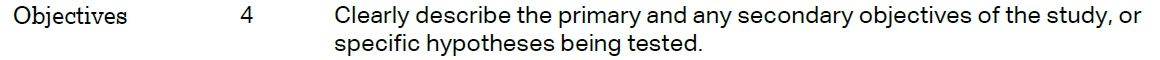 | | | page 5 |  |
| METHODS | | |  |  |
| 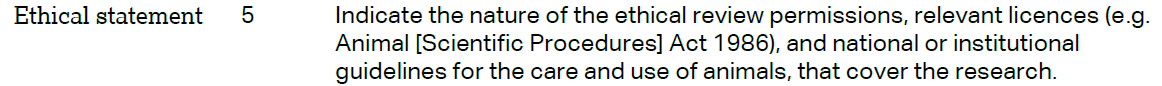 | | | page 7-8 |  |
| 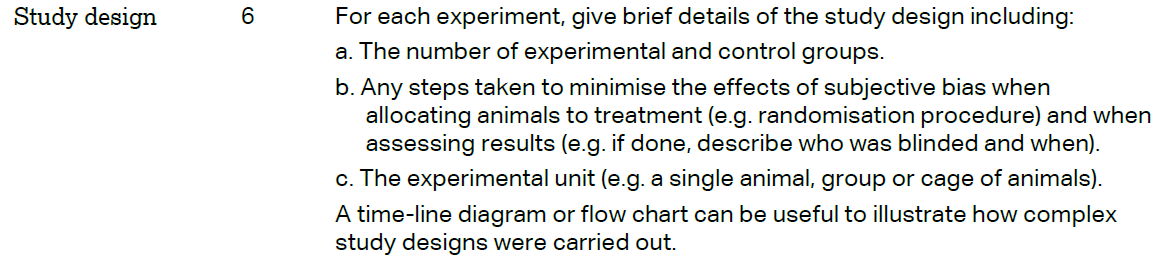 | | | pages 8 - 10 |  |
| 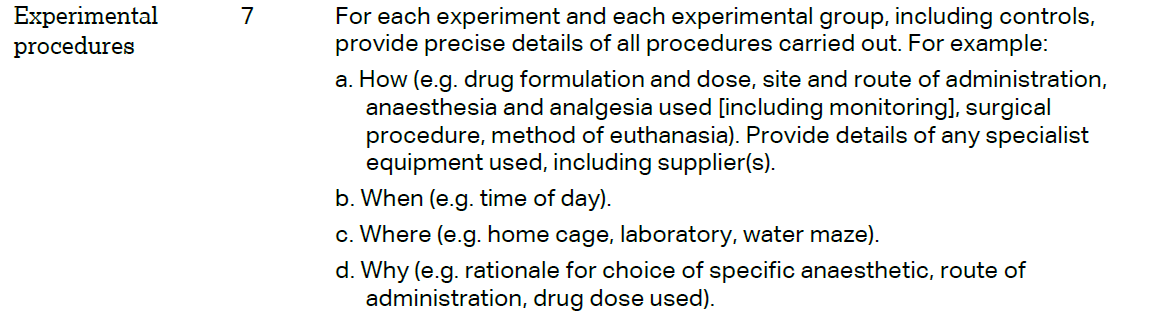 | | | page 8 |  |
| 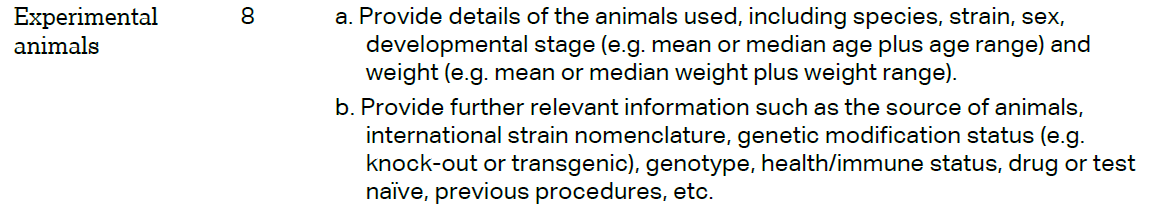 | | | page 7 |  |

The ARRIVE guidelines. Originally published in *PLoS Biology*, June 2010^1^

| 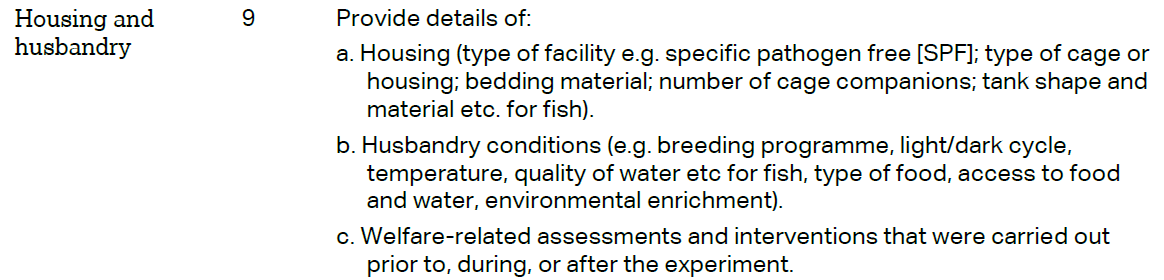 | page 7 | |
| --- | --- | --- |
| 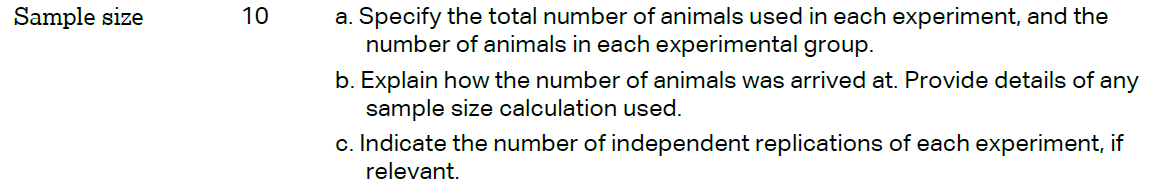 | pages 9 - 10 | |
| 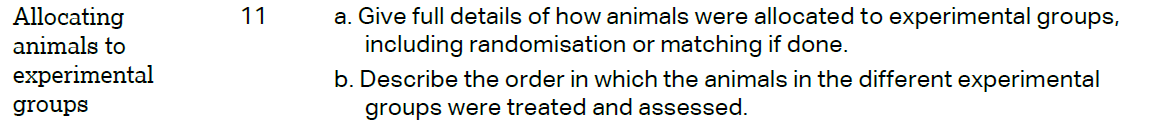 | ND | |
| 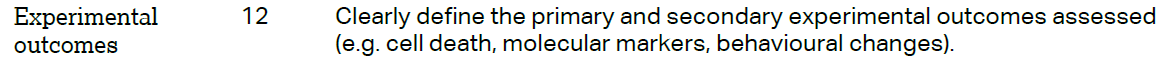 | pages 9 - 10 | |
| 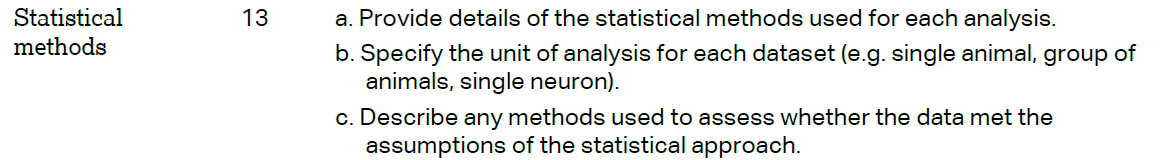 | page 11 | |
| RESULTS |  | |
| 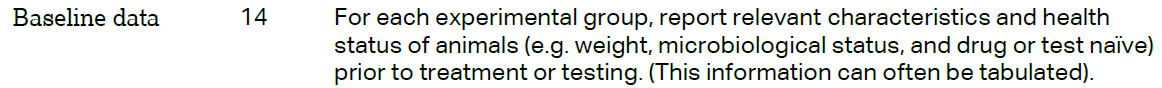 | page 12 | |
| 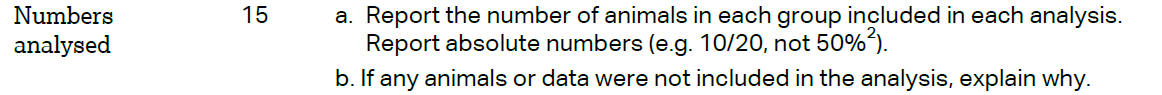 | page 12 | |
| 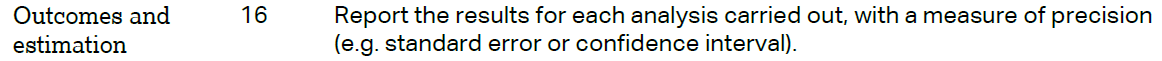 | pages 12-14 | |
| 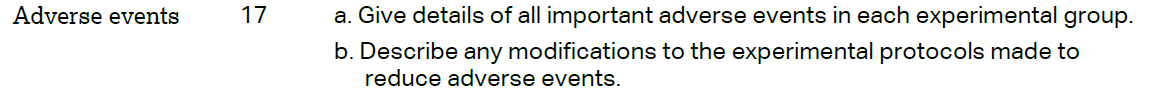 | ND | |
| DISCUSSION |  | |
| 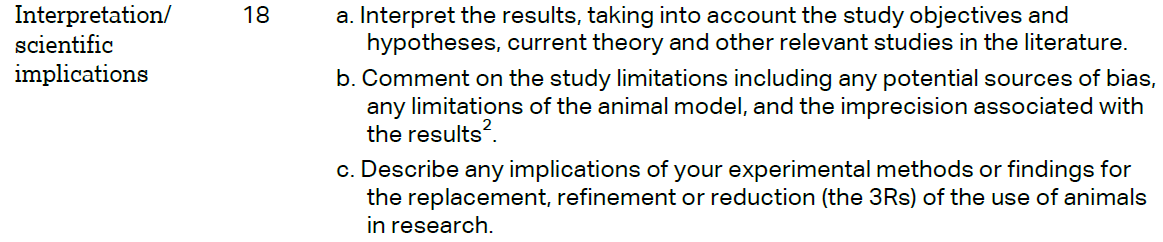 | pages 14 - 18 | |
| 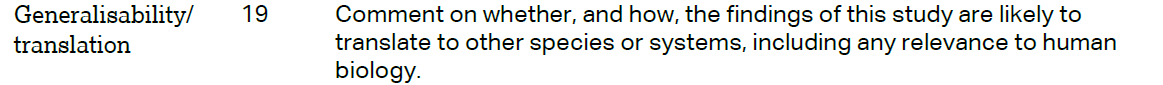 | page 18 - 19 | |
| 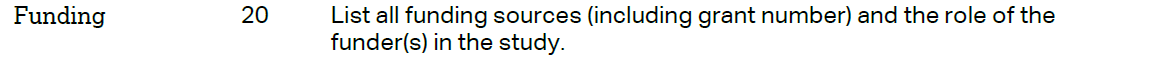 | | not to be included in the manuscript for PlosOne |


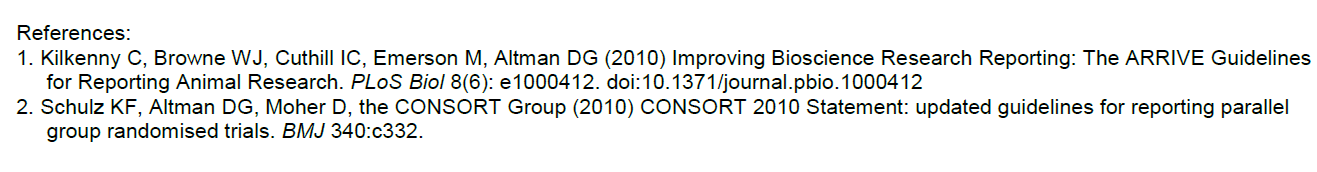

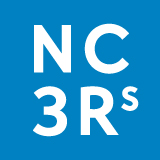

Supplement: S1 File — (DOCX) [file pone.0134378.s001.docx]
